# Agronomic management drives the wheat yield plateau in high-yielding environments of northwest Europe

**DOI:** 10.1038/s43016-025-01286-w

**Published:** 2026-01-20

**Authors:** João Vasco Silva, Bert Rijk, Herman N. C. Berghuijs, Allard J. W. de Wit, Pytrik Reidsma, Martin K. van Ittersum

**Affiliations:** 1 Plant Production Systems, Wageningen University, Wageningen, The Netherlands; 2 Sustainable Agrifood Systems, International Maize and Wheat Improvement Center, Harare, Zimbabwe; 3 Plant Production in the Tropics and Subtropics, University of Hohenheim, Stuttgart, Germany; 4 Earth Observation and Environmental Informatics, Wageningen Environmental Research, Wageningen, The Netherlands

**Keywords:** Agriculture, Climate sciences

## Abstract

Northwest Europe experienced considerable increases in wheat yield until the mid-1990s, but progress has remained stagnant since then. Estimating the relative contributions of improved genetics, historical climate change and agronomic management to this yield plateau is required to understand the feasibility of yield increases in the future. Analysis of high-quality experimental data revealed yield gains due to improved genetics of 74–84 kg ha^−1^ yr^−1^ during the period 1994–2016. Thus far, yield gains due to historical climate change of 26–60 kg ha^−1^ yr^−1^ were estimated over the same period using a well-validated crop model across regions, soil types and cultivars. Given the absence of genetic and climatic yield ceilings, we conclude that agronomic management is responsible for the wheat yield plateau in northwest Europe, contributing to unrealized potential yield gains of 67–114 kg ha^−1^ yr^−1^. Breaking the yield plateau will require due attention to agronomic constraints at the farm level and continued monitoring of genetic gains and climate change impacts on wheat yields.

## Abstract (web summary)

Wheat yields in northwest Europe have plateaued since the mid-1990s. This study finds that no ceiling in genetic yield potential has been reached and that climatic conditions have not constrained wheat yields across high-yielding environments in the region thus far; suboptimal agronomic management is responsible for unrealized wheat yield progress of 67–114 kg ha^−1^ yr^−1^ during the period 1994–2016.

## Main

**Fig. 1:**
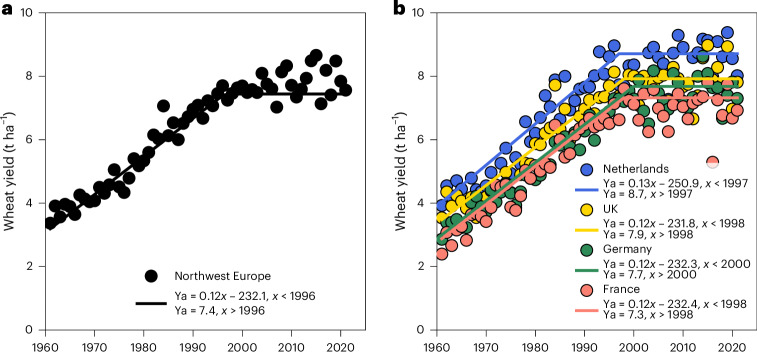
Actual wheat yield progress and plateaus in northwest Europe. **a**,**b**, The solid lines are linear regressions with an upper plateau fitted to the data, indicating stagnation in wheat yield in northwest Europe (**a**) and selected countries (**b**). Wheat yields are reported at commercial dry matter concentrations. Data source: FAOSTAT. Source data

Northwest Europe is an important breadbasket with Germany, France, the UK, the Netherlands, Belgium and Denmark cultivating about 10 Mha of wheat annually and being responsible for nearly 10% of the world’s wheat production (FAOSTAT statistical database, https://www.fao.org/faostat/, 2023). Considerable wheat yield progress, at a rate of 120 kg ha^−1^ yr^−1^, was observed in the region between 1961 and the mid-1990s, after which a yield plateau was reached at 7.4 t ha^−1^ (Fig. 1a). Progress in wheat yield remained stagnant since then, a trend also evident across individual countries^1–3^ (Fig. 1b). For instance, wheat yield in the Netherlands increased by 130 kg ha^−1^ yr^−1^ between 1961 and 1997, plateauing at 8.7 t ha^−1^ since then (Fig. 1b). Wheat yield in the UK, Germany and France increased by 120 kg ha^−1^ yr^−1^ up to the late 1990s, followed by a period of no further wheat yield progress with a plateau at 7.9 t ha^−1^, 7.7 t ha^−1^ and 7.3 t ha^−1^, respectively (Fig. 1b).

Identifying the mechanisms responsible for yield plateaus is critical to delineate the feasibility of yield increases in the future. Crop yield is a function of genotype by environment by management interactions (G × E × M)^4^. In this context, it is helpful to differentiate four yield levels. The potential yield (Yp) refers to the yield of a cultivar when grown with non-limiting availability of water and nutrients and biotic stresses are effectively controlled^5^. The water-limited potential yield (Yw) is defined similarly to Yp, but it considers that crop growth can be limited by water supply. The water- and nitrogen-limited yield (Ywn) is defined similarly to Yw, but also considers nitrogen (N) limitations during the growing season^6^. Finally, the actual yield (Ya) refers to the yield achieved by farmers. The yield gap (Yg) is then defined as the difference between Yp (irrigated conditions) or Yw (rain-fed conditions) and Ya^5^ and indicates the scope to increase crop yield on existing cropland.

Plateaus in actual yields can occur over time when farm management practices are not able to exploit potential yields and/or due to ceilings in potential yields. The latter can be explained by climatic constraints on Yp (or Yw) and/or by ceilings in genetic yield potential (as reported for rice and maize^7^). Analyses of cultivar trials under optimal growth conditions showed linear increases in genetic yield potential for wheat in most northwestern European countries^8^. For instance, genetic progress of about 100 kg ha^−1^ yr^−1^ (1978–2016) was reported in the Netherlands^9^. Similar increases were reported in France (94–128 kg ha^−1^ yr^−1^ between 1970 and 2008^1^), Germany (55 kg ha^−1^ yr^−1^ between 1983 and 2014^10^), the UK (70 kg ha^−1^ yr^−1^ between 1948 and 2007^11^) and other parts of northern Europe^12^. Genetic progress has been attributed to increases in thermal time from anthesis to maturity, advancing the grain-filling period closer to the longest days of the year, and increases in reference light use efficiency^13,14^. Existing evidence thus indicates that a yield ceiling in genetic yield potential is probably not responsible for the wheat yield plateau in northwest Europe.

Given the increases in genetic yield potential, environmental and/or management factors must then explain the wheat yield plateau^1,15,16^. Historical trends in growing-season temperature and rainfall were shown to explain 10% of the slowdown in wheat and barley yields across Europe^17^, with the authors speculating that agro-environmental policies would be responsible for the yield plateau. These results were partly confirmed by a study^18^ showing that historical climate change affected wheat yields in Europe negatively by 2–9%. It has also been shown that wheat yield trends in Europe were less positive where temperature was increasing faster and where wheat area shares were greater^19^. Northwest Europe also experienced substantial changes in nutrient use during the past decades^20^ (Supplementary Fig. 5), and indeed, farmers’ management practices have been influenced by environmental policies.

Understanding the driving forces behind the wheat yield plateau in northwest Europe is important to global food availability, given global increases in wheat demand, and more so in the context of future climate change. Our study disentangles the contribution of genetic improvement, historical climate change and agronomic management to the wheat yield plateau in northwest Europe during the past half century. This was achieved by combining experimental data from cultivar tests, wheat experiments and crop simulation modelling for high-yielding environments in northwest Europe, where wheat is a rotation crop for other cash crops. Because agronomy is relatively well developed in these regions, our results provide a conservative estimate of its contribution to wheat yield trends. Insights from our analysis are important for arable farming systems in Europe, where wheat will remain an important crop, and for other breadbaskets affected by yield plateaus now and in the future.

## Results

### Yield gains due to genetics, climate change and agronomy

**Fig. 2:**
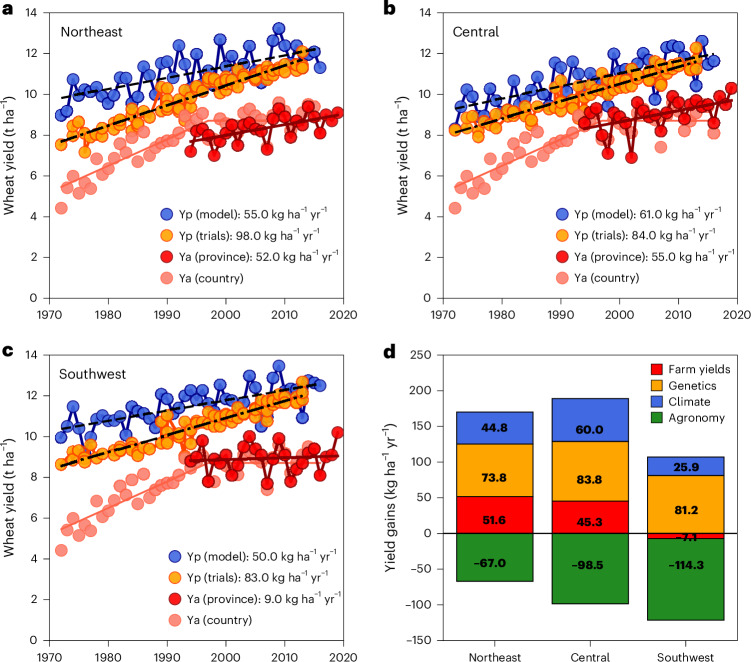
Yield gains due to genetic improvement, historical climate change and agronomic management in high-yielding environments of the Netherlands. **a**–**c**, Key production regions include the northeast (**a**), central (**b**) and southwest (**c**) regions of the Netherlands. Ya at regional and national levels was obtained from the National Statistics Bureau of the Netherlands. Linear fits to the best linear unbiased estimates for individual cultivars in official variety trials provide a proxy for yield gains due to genetic improvement, and linear fits to the Yp simulated with the crop model WOFOST for a modern wheat cultivar released in 2009 provide a proxy for yield gains due to historical climate change. Values in the legend refer to yield gains per year. Yield gains due to historical climate change and agronomic management under water-limited conditions and for old and modern cultivars are provided in Supplementary Table 3. **d**, Yield gains estimated for the 1994–2016 period. All directly estimated trends for the 1994–2016 period were statistically significant (*P* ≤ 0.05), except for the actual yield trend in the central region (*P* ≤ 0.10) and the actual and climate yield trends in the southwest region (*P* > 0.10). Trends in nitrogen recommendations for wheat in the case study regions are provided in Supplementary Fig. 4. Source data

Yield records from official cultivar trials conducted under optimal growth conditions were used to estimate the yield gain due to genetic improvement between 1972 and 2016. Genetic progress in yield potential in these trials was 98, 84 and 83 kg ha^−1^ yr^−1^ for the case study regions (Fig. 2). Wheat yield at the start of the trials in 1972 was about 8 t ha^−1^ and reached 12 t ha^−1^ after 2010, pointing to a 50% increase in genetic yield potential over 40 years. Yield gains due to genetic improvement were smaller but significantly positive, 74–84 kg ha^−1^ yr^−1^, during the period 1994–2016 (Fig. 2d).

A well-validated crop model (Supplementary Table 1) was used to simulate long-term changes in Yp (and Yw) for a modern cultivar assuming constant agronomic management. Trends in simulated yields thus provide the unbiased contribution of historical climate change to yield progress. Historical climate change had a positive impact on Yp between 1972 and 2016, contributing to yield gains of 50–61 kg ha^−1^ yr^−1^ in the case study regions (Fig. 2). The simulated Yp for the modern cultivar Julius (released in 2009) increased from 8–10 t ha^−1^ in the 1970s to 11–13 t ha^−1^ after the year 2000. The genetic yield potential and the simulated Yp converged and were similar since 2010 (Fig. 2), which was about the year of release of the cultivar used in the model simulations. Yield gains due to historical climate change were also positive when considering the 1994–2016 period only: 45 kg ha^−1^ yr^−1^ in the northeast region (*P* ≤ 0.10), 60 kg ha^−1^ yr^−1^ in the central region (*P* ≤ 0.05) and 26 kg ha^−1^ yr^−1^ in the southwest region (trend not statistically different from 0; Fig. 2d).

Yield gains due to historical climate change were also estimated without CO_2_ fertilization effects (Supplementary Fig. 3), for an old cultivar and for different soil types under water-limited conditions (Supplementary Table 3). Yield gains without CO_2_ fertilization were smaller relative to those estimated with CO_2_ fertilization, but non-negative in all three regions (Supplementary Fig. 3). This is important because yield gains without CO_2_ fertilization provide an absolute lower bound for the effect of climate change on wheat yield. The old cultivar Arminda (released in 1977) showed yield gains due to historical climate change (1972–2016) of 4–11 kg ha^−1^ yr^−1^ higher than those estimated for the modern cultivar. No major differences in yield gains due to historical climate change were observed between simulations conducted under potential and water-limited situations in clay soils (<2 kg ha^−1^ yr^−1^ across regions and cultivars), whereas for sandy soils, yield gains were 7 and 13 kg ha^−1^ yr^−1^ lower than those estimated under potential production. Although CO_2_ fertilization, cultivar choice and drought stress affected the yield gains due to historical climate change, the trend of their impacts on past yield progress confirms the positive effect of historical climate change on wheat production in high-yielding environments of northwest Europe.

Progress in farm yields was observed in two of the three regions, namely northeast and central Netherlands (52–55 kg ha^−1^ yr^−1^), but not as much in southwest Netherlands (9 kg ha^−1^ yr^−1^), where a clear yield plateau was observed since the mid-1990s (Fig. 2). Such progress in wheat yields was accompanied by increases in recommended fertilizer rates since the 1980s, from 80–100 kg N ha^−1^ up to 200–220 kg N ha^−1^ in recent years (Supplementary Fig. 5). Given the positive yield gains due to genetic improvement and historical climate change, it follows that an unrealized yield gain of 67–114 kg ha^−1^ yr^−1^ (1994–2016) can be attributed to suboptimal agronomic management in high-yielding environments of northwest Europe (Fig. 2d).

### Effect of historical climate change on potential yields

Historical weather data and crop model simulations for a modern cultivar were used to explain variability in Yp trends. Cumulative seasonal radiation during the reproductive stage increased by 1.459 and 1.871 MJ m^−2^ yr^−1^ in the southwest and central regions between 1972 and 2016 (Table 1). There were statistically significant increases in average maximum and average minimum temperature during the growing season and during the vegetative stage in all regions (Table 1). Increases in average maximum temperature ranged between 0.031 and 0.037 °C yr^−1^, whereas increases in average minimum temperature ranged between 0.023 and 0.029 °C yr^−1^. Statistically significant increases in minimum average temperature during the reproductive stage (0.027 °C yr^−1^) were observed in only one of the case study regions (northeast). Finally, no statistically significant changes in cumulative rainfall were detected in either region, independent of crop growth stage (Table 1).

**Table 1:** Historical climate change across high-yielding environments for wheat in the Netherlands between 1972 and 2016

Variable	Period	Eelde (northeast)	De Bilt (central)	Vlissingen (southwest)
Cumulative radiation (MJ m^−2^ yr^−1^)	Growing season	−0.433, NS	0.976, NS	0.816, NS
	Vegetative	−1.087, NS	−1.008, NS	−0.693, NS
	Reproductive	0.753, NS	1.871^a^	1.459^a^
Average *T*_max_ (°C yr^−1^)	Growing season	0.032^a^	0.035^a^	0.031^a^
	Vegetative	0.033^a^	0.037^a^	0.033^a^
	Reproductive	0.032, NS	0.026, NS	0.022, NS
Average *T*_min_ (°C yr^−1^)	Growing season	0.025^a^	0.023^a^	0.026^a^
	Vegetative	0.025^a^	0.026^a^	0.029^a^
	Reproductive	0.027^a^	0.008, NS	0.016, NS
Cumulative rainfall (mm yr^−1^)	Growing season	0.118, NS	0.767, NS	0.298, NS
	Vegetative	−0.406, NS	0.055, NS	0.132, NS
	Reproductive	0.505, NS	0.698, NS	0.215, NS

Cumulative solar radiation, average maximum air temperature (*T*_max_), average minimum air temperature (*T*_min_) and cumulative rainfall were computed for the entire wheat-growing season and for the vegetative and reproductive stages. WOFOST-simulated phenological stages for a single modern wheat cultivar were used to summarize the weather data for the different crop growth periods: growing season, from emergence to maturity; vegetative stage, from emergence to anthesis; and reproductive stage, from anthesis to maturity. ^a^Regression slope statistically significant at the 5% level. NS, regression slope not statistically significant.

**Fig. 3:**
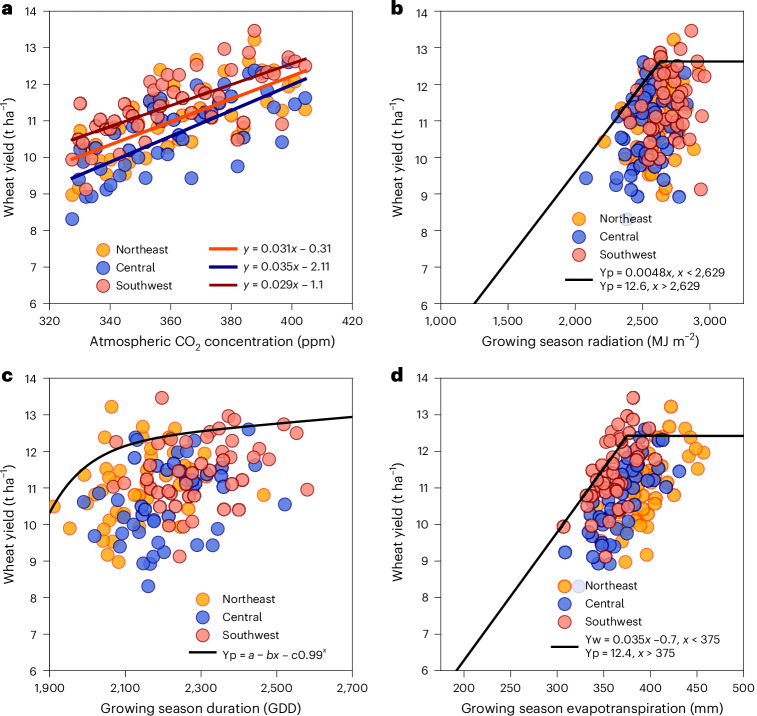
Wheat Yp response to climatic conditions across high-yielding environments in the Netherlands simulated with the WOFOST crop model for a modern wheat cultivar. **a**–**d**, Climatic conditions include atmospheric CO_2_ concentration (**a**), solar radiation (**b**), growing degree days (**c**) and evapotranspiration (**d**). Simulations assumed constant genetics and agronomic management throughout the simulation period (Methods). Solid lines show linear regressions fitted to the data (**a**) and linear-plateau boundary functions fitted to the 90th quantile of the data (**b**–**d**). Note that the *y*-axis starts at 6 t ha^−1^ in all panels. GDD, growing degree days. Source data

Yp increased linearly with increases in atmospheric CO_2_ concentration, at a rate of 29–35 kg ha^−1^ ppm^−1^ (Fig. 3a), confirming the importance of CO_2_ fertilization to yield gains due to historical climate change (Supplementary Fig. 3). The relationship between Yp and seasonal radiation (Fig. 3b), cumulative growing degree days (Fig. 3c) and seasonal evapotranspiration (Fig. 3d) was described by linear-plateau boundary functions fitted to the 90th quantile of the data. Yp responses to seasonal radiation were observed up to 2,600 MJ m^−2^, after which no yield response was observed. Similarly, relationships between Yp and growing degree days and evapotranspiration were observed up to 2,100 °C per day and 375 mm, respectively. Wheat yield across most site × year combinations was probably not limited by solar radiation, growing degree days or evapotranspiration.

**Fig. 4:**
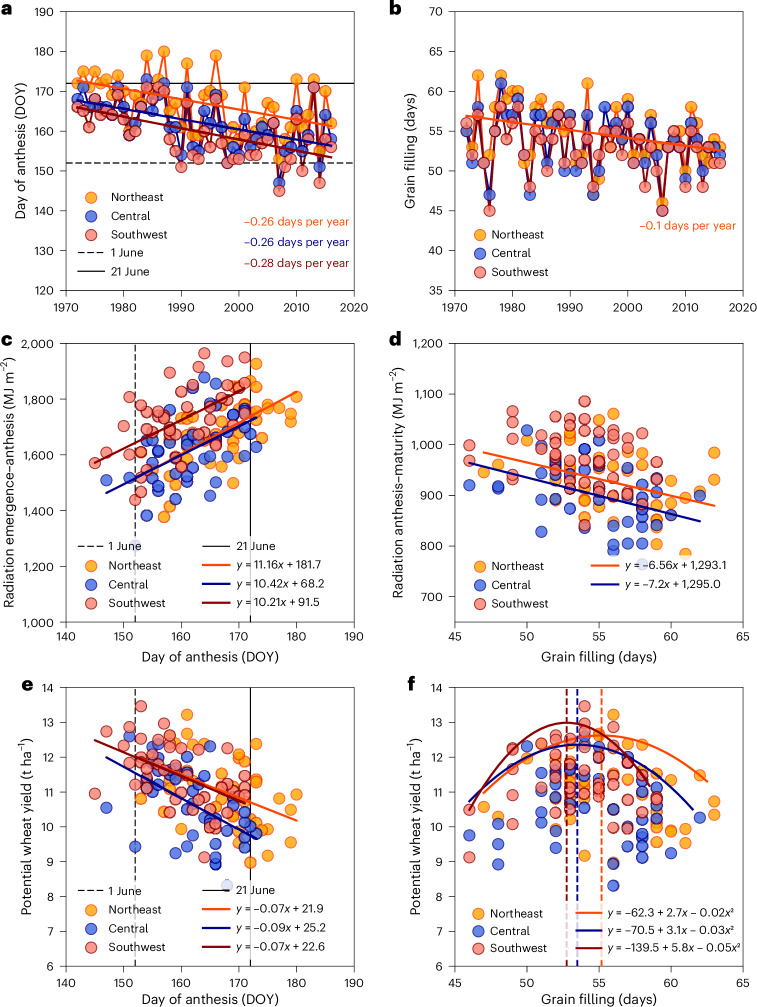
Effect of historical climate change (1972–2016) on wheat development and radiation-use efficiency across high-yielding environments in the Netherlands simulated with the WOFOST crop model for a modern cultivar. Simulations assumed constant genetics and agronomic management throughout the simulation period (Methods). **a**–**f**, Data show changes in anthesis dates and number of grain-filling days (**a**,**b**), cumulative solar radiation during vegetative (emergence–anthesis) and reproductive stages (anthesis–maturity) in relation to the anthesis date and number of grain-filling days (**c**,**d**) and wheat Yp in relation to anthesis date and number of grain-filling days (**e**,**f**). Solid lines in **a**–**e** and **f** show linear regressions with slope statistically different from 0 (*P* < 0.05) and quadratic boundary functions fitted to the 90th quantile of the Yp data, respectively. Note that the *y*-axis in **e** and **f** starts at 6 t ha^−1^ and that fitted regression lines partially overlap for the central and northeast regions in **c** and northeast and southwest regions in **e**. DOY, day of the year. Source data

Past increases in air temperature during the vegetative stage resulted in earlier anthesis dates over time, an advance of 0.26–0.28 days per year (Fig. 4a). In the 1970s, simulated anthesis dates for the modern cultivar were close to 21 June, the longest day of the year. Yet, those were advanced to dates closer to 1 June after 2010. Conversely, a reduction in the number of grain-filling days over time was observed (Fig. 4b), but the fitted regression was only statistically significant for the region where significant increases in minimum air temperature during the reproductive stage were observed (Table 1). Anthesis dates closer to 1 June were associated with less solar radiation during the vegetative stage (Fig. 4c), more radiation during the grain-filling period (Fig. 4d) and greater Yp in all regions (Fig. 4e) compared with anthesis dates closer to 21 June. Conversely, solar radiation during the reproductive stage was negatively associated with the number of grain-filling days in two of the case study regions (Fig. 4d). These results can be explained by increases in solar radiation during the reproductive stage (Table 1) and the number of grain-filling days varying little around the plateaus of the curves (Fig. 4f).

### Yield gaps and constraints due to agronomic management

A yield gap analysis was conducted for 141 field–year combinations in Flevoland, central Netherlands, to unpack the contribution of water, N and other management constraints to wheat yields on-farm. This analysis focused on this high-productivity region^21^ owing to its favourable environment such that farm yields close to the genetic and climatic potential can be expected. As sound agronomy is well established and widely adopted in this region, our results offer a lower bound for the importance of agronomic constraints compared with other production environments in Europe where agronomic management might not be as optimal and extreme weather events more frequent.

**Fig. 5:**
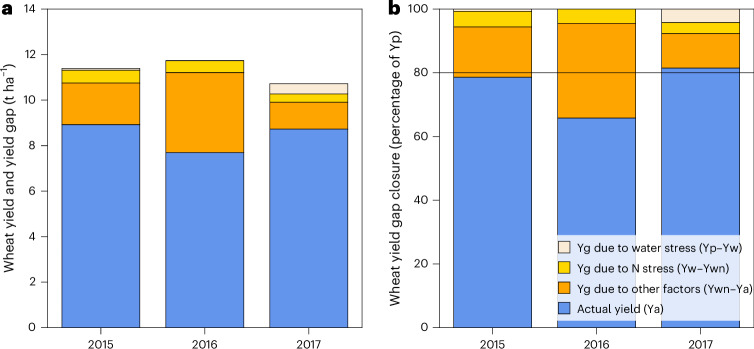
On-farm yields and yield gaps in Flevoland, central Netherlands, a high-yielding favourable environment for wheat production in northwest Europe. Yp, Yw and Ywn were simulated with the WOFOST crop model for 141 field–year combinations as described elsewhere^6^. Actual yields were obtained from ref. ^21^. **a**,**b**, Yields and yield gaps (Ygs) are presented in absolute terms (**a**) and relative to Yp (**b**). The difference between Yp and Yw captures Ygs owing to water stress. The difference between Yw and Ywn captures Ygs owing to N stress. The difference between Ywn and Ya captures Ygs due to management factors unrelated to water and N. The N management practices reported by farmers and the respective N use efficiency are provided in Supplementary Fig. 4. Source data

The simulated Yp, Yw and Ywn were, on average (±s.d.), 11.4 ± 0.6, 11.2 ± 0.7 and 10.7 ± 1.1 t ha^−1^ across field–year combinations (Fig. 5), whereas the farmer-reported Ya was, on average, 8.5 ± 0.9 t ha^−1^ (Fig. 5). Water stress was noticeable in 67 field–year combinations with an average Yg between Yp and Yw of 0.1 t ha^−1^ (about 2% of Yp; Fig. 5). N stress was noticeable in 99 field–year combinations with an average Yg between Yw and Yn of 0.5 t ha^−1^ or about 5% of Yp. N input (above 207 kg N ha^−1^) was indeed high enough in most of the field–year combinations to reach Yp (Supplementary Fig. 5). Other factors besides water and N availability were responsible for an average Yg of 2.3 t ha^−1^, or 20% of Yp. Management practices unrelated to water and N were therefore the most important constraints to wheat yields in this high-yielding environment of northwest Europe.

## Discussion

Disentangling the contribution of genetic improvement, historical climate change and agronomic management to actual yield trends is critical to understand the feasibility of yield increases in the future^22,23^. This is of particular importance in northwest Europe, an important breadbasket where little yield progress has been observed since the mid-1990s^1,15^. Our results provide new evidence that no ceiling in genetic yield potential has been reached and that climatic conditions have not constrained wheat yields in the region thus far; hence, suboptimal agronomic management drives the observed yield plateau (Fig. 2). Despite some regional differences in agronomic management and extreme weather events, our findings have wider relevance owing to similarities in climatic conditions, genetic gains and production potential between our case study regions and other wheat-producing areas in northwest Europe also experiencing a yield plateau^24^.

Our integrated approach to estimate yield gains due to agronomic management relied on the difference method^22^, which makes our estimates sensitive to uncertainties in the yield gains attributed to genetic improvement and historical climate change. We also did not account for interactions between genetics and environment on the one hand and agronomy on the other^4,25^ owing to data limitations. A two-step statistical approach controlling for year-specific fixed effects attributed to changes in climate and agronomy over time was used to estimate unbiased yield gains due to genetic improvement in cultivar trial data^9^. These ranged between 74 and 84 kg ha^−1^ yr^−1^ (1994–2016), in agreement with other studies^1,8,10,11^. The database used to quantify genetic progress in Yp included a total of 84 cultivars over four decades, translating into a high turnover of lower-performing cultivars by more recent, better-performing ones, which is essential to measure yield potential^4,25^. Our estimates of genetic progress are therefore unlikely to challenge the importance of agronomic management to the wheat yield plateau in northwest Europe.

The effect of historical climate change on wheat yield was assessed with a well-validated crop model^6,13^, as opposed to earlier statistical assessments of yield data aggregated across large scales^17–19^. We found that historical climate change had a positive impact on Yp and Yw, being responsible for yield increases of 26–60 kg ha^−1^ yr^−1^ (1994–2016) across regions, soil types and cultivars. Historical climate change benefited wheat production particularly through increases in atmospheric CO_2_ concentration but also through increases in solar radiation during grain filling. The effect of CO_2_ fertilization on wheat yield is well established^26^, yet future studies are required to assess the effect of interactions between CO_2_ and N fertilization effects on wheat yield trends. Our results also showed that increases in solar radiation during grain filling were associated with earlier anthesis dates owing to increases in temperature, such that grain filling occurred around days with the highest daily radiation in northern latitudes. Our simulations also revealed a G × E interaction on sandy soils, implying that the old cultivar benefited slightly more from historical climate change, although its yields were below those of the modern cultivar. It remains to be seen whether future temperature increases might further reduce the duration and advance the timing of the grain-filling period such that it continues affecting wheat yield positively^27,28^.

Our assessment of yield gains due to historical climate change is limited by the inability of crop models to simulate the impacts of extreme weather events on crop yields^21,29,30^. Two important weather extremes for arable crops in the Netherlands are an extreme dry growing period and a wet harvesting period^31^. Yet, no relationship between these and yield anomalies was found for winter wheat^31^. These findings align with our assessment that extreme weather events have had a small impact on wheat yield (Supplementary Fig. 4). The same might not apply to other wheat-growing areas of northwest Europe, particularly where context- and year-specific yield losses due to extreme weather were documented^32,33^. A combination of model-based and experimental approaches is needed to further assess the effect of extreme weather events on wheat yield^34^ and how projected climate change may impact these across northwest Europe in the future.

Given the absence of genetic and climatic yield ceilings, suboptimal agronomic management is thus responsible for an unrealized yield progress of 67–114 kg ha^−1^ yr^−1^ (1994–2016). These are probably conservative estimates as our analyses focused on high-yielding environments where agronomic management is close to optimal. Despite the agro-environmental policies in place, it is unlikely that crop nutrition constrained wheat yields^35,36^, as also confirmed in our reanalysis of farmer field data (Supplementary Fig. 5). Indeed, current N application rates for wheat are similar to or greater than the minimum N requirements to reach 80% of Yw for most countries in northwest Europe (www.yieldgap.org)^37^, and N rates on-farm are comparable to those recommended at regional level and often above that required to achieve Yp (Supplementary Fig. 5). P and K probably did not limit wheat yield either, despite lower applications over time, owing to residual effects from past applications^38^. The same is true for water as earlier studies found increasing trends for Yp and Yw and non-significant changes in seasonal rainfall^21,24^. The impact of drought stress varies within the region though^24,39^, with the Netherlands being one of the least affected^24^; hence, water-related constraints might explain (part of) the wheat yield plateau in areas with more variable rainfall and light-textured soils. This is in stark contrast to findings from dryland regions where seasonal rainfall is the primary determinant of wheat productivity^40^.

Wheat is a secondary, rotational crop relative to cash crops^41^ in our study regions as opposed to other regions where it is a main crop for farmers. This has implications for crop management as crop rotations optimized for high-value root and tuber crops often lead to soil compaction^42^, relatively late planting^21^ and soil-borne diseases^43^, all detrimental factors to wheat productivity. Other factors may be constraining wheat yield as well, including fungal diseases and the timeliness of operations, owing to less careful management in peak labour periods. Disease pressure is projected to further increase under future climate change. Most important perhaps is that wheat remains a relatively low-value crop in, for example, the Netherlands and Belgium, and its cultivation needs to comply with environmental regulations limiting input use and farm management more broadly.

Realizing the untapped wheat yield gains in northwest Europe will be important for global food security under climate change given the increasing wheat demand and the importance of the crop in current cropping systems. Yet, this will probably be more challenging in future owing to possible ceilings in genetic yield potential, as recently reported for the first time in Germany^44^, and by new cultivars not being able to benefit from future climate change owing to further shortening of the growing season and the increased frequency of extreme weather events. And above all, yield increases will require a conducive environment for sustainable wheat production at the farm level so that farmers prioritize wheat over more profitable crops and policy impacts on agronomic management and farm performance are evaluated ex ante.

## Methods

Different methods and sources of data were used to disentangle the contribution of improved genetics, historical climate change and agronomic management to the wheat yield plateau in northwest Europe (Supplementary Table 2). Trends in actual farm yields depict the full interaction between genotype, environment and management and were estimated from official statistics using linear regression. Yield gains due to genetic improvement capture cultivar characteristics conferring higher yield potential and were estimated from cultivar trials using a two-step regression approach. Yield gains due to historical climate change isolate the effects of seasonal radiation, temperature, rainfall, evapotranspiration and atmospheric CO_2_ concentration to crop yield and were estimated through a combination of crop simulation modelling and linear regression. Lastly, yield gains due to agronomic management were estimated with the difference method and capture field- and farm-level factors affecting yield losses to water and nutrient stress and pest, disease and weed pressure.

### Genetic improvement

Wheat yield data from official cultivar trials conducted under the auspices of the Applied Plant Research Institute in the Netherlands between 1970 and 2016 were used to estimate genetic progress in yield potential^9^. Cultivar trials were conducted under optimum nutrient and crop protection management. Although these trials were not irrigated, we expected little water stress given the relatively even rainfall during the growing season, good soil profile and high groundwater levels resulting in capillary rise. Therefore, wheat yields in these trials can be considered an experimental proxy for Yp.

Data analysis was conducted separately for three case study regions representing high-yielding environments for wheat production in northwest Europe. Estimation of genetic progress in yield potential was done in two steps^9^, using the lm() and emmeans() functions in R (ref. ^45^). First, a linear regression model with measured wheat yield at 85% dry matter content as the dependent variable and cultivar and harvest year as independent variables was fitted to remove year effects associated with changes in climate and/or management practices from the genetic contribution to yield progress. The best linear unbiased estimates for individual cultivars obtained with this model were further regressed against the year of release of each cultivar (defined as the first year a cultivar was included in the trials). The slope of this regression indicates the yield gain due to genetic improvement, and it was estimated for the periods 1972–2016 and 1994–2016. This two-step regression approach provides an unbiased estimate of genetic gain in yield potential as it controls for changes in environmental conditions and agronomic management in the cultivar trials over time.

### Historical climate change

The World Food Studies (WOFOST) crop model (v8.1) as implemented in the Python Crop Simulation Environment^46^ was used in this study. WOFOST is a semi-deterministic crop growth simulation model of physiological processes, including crop phenology, light interception, photosynthesis, respiration, assimilate partitioning, leaf area dynamics and evapotranspiration. WOFOST simulates crop production under potential and water-limited growth conditions with a daily time step^46^, and it was recently extended to simulate water- and nitrogen-limited growth^6^. The extended model was recalibrated and evaluated for old and modern wheat cultivars against high-quality experimental data, as summarized in Supplementary Information.

The WOFOST crop model was used to simulate Yp and Yw for the regions, and time span, where the cultivar trials were conducted. Simulations considered one set of crop parameters (cultivar Julius, released in 2009) between 1972 and 2016. In doing so, simulated yields do not consider effects of climate change adaptation due to genetic improvement, which is important given our objective of disentangling the contribution of historical climate change from that of genetic improvement and agronomic management to yield trends. Yet, as yield gains due to historical climate change are cutlivar dependent, we conducted a similar set of simulations for an old cultivar (Arminda, released in 1977) and assessed how cultivar choice and G × E interactions impacted the estimated yield gains (Supplementary Table 2). Two contrasting soil types in terms of water-holding capacity, clay and sandy, were considered for the Yw simulations. The soil parameters for these soil types were obtained by estimating the van Genuchten parameters^47^ for a representative clay soil and a representative sandy soil from the Dutch soil map BOFEK^48^ followed by the conversion of these parameters to WOFOST input parameters^6^. The sowing date was not recorded in the cultivar trials; hence, it was set at 15 November each year, and the simulations used observed daily weather data from three weather stations maintained by the Royal Netherlands Meteorological Institute, namely, Eelde (53.125° N, 6.585° E; representative for region northeast), De Bilt (52.100° N, 5.180° E, central) and Vlissingen (51.442° N, 3.596° E, southwest). These weather stations were selected considering their proximity to the experimental sites and the number of years with available records. Daily observations of solar radiation (kJ m^−^^2^ per day), minimum and maximum air temperature (^∘^C), rainfall (mm per day) and wind speed (m s^−1^) were directly available for each weather station and analysed for changes in extreme weather events during the wheat-growing season over time (Supplementary Fig. 4). Daily vapour pressure was estimated from the minimum temperature, and atmospheric CO_2_ concentration was obtained from the National Oceanic and Atmospheric Administration. Simulations were also conducted at standard CO_2_ concentrations of 360 ppm and 400 ppm to assess the effect of CO_2_ fertilization on wheat yields (Supplementary Fig. 3).

Yield gains due to historical climate change were estimated from the crop model outputs as the slope of the linear regression between the simulated yields and the respective harvest year considering the periods 1972–2016 and 1994–2016. Crop model simulations were conducted for the old and the modern cultivar under potential (Yp) and water-limited (Yw) conditions on a clay and sandy soil (Supplementary Table 3). This allowed us to assess the effect of drought stress and cultivar type on the yield gains due to historical climate change and on G × E interactions affecting them. Trends in Yp for the modern cultivar are presented in Fig. 2 as potential growth conditions are justified in the case study regions^24^. Trends for the old cultivar under potential conditions and the old and modern cultivars under water-limited conditions are presented in Supplementary Table 3.

Crop model outputs for the case study regions were further analysed to understand the effect of historical climate change on Yp. First, trends in historical climate change (1972–2016) regarding cumulative radiation, average maximum and minimum air temperature and cumulative rainfall for the entire growing season (that is, number of days between emergence and maturity) and for the vegetative and reproductive stages were quantified for each region using linear regression. Growth stages in this analysis were simulated with WOFOST, considering a single set of crop parameters for the modern cultivar throughout the simulation period. Second, Yp response to atmospheric CO_2_ concentration was quantified using linear regression whereas nonlinear boundary functions fitted to the 90th quantile of the data (with the nlrq() function of the R package quantreg^49^) were used to characterize Yp response to seasonal radiation, growing degree days and evapotranspiration. Finally, temporal changes in the date of anthesis and the grain-filling days were quantified using linear regression and these variables were further related to cumulative radiation during the vegetative and reproductive stages, respectively, and Yp. Boundary functions were fitted to the pooled data, whereas linear regression analyses were region specific. Linear regressions were shown only if the respective slope was significantly different from zero at 5% significance level.

### Agronomic management

Yield gains under on-farm conditions were estimated as the slope of the linear regression between Ya from official statistics at the regional level and the respective harvest year. Yield gains due to agronomic management were then estimated as the difference between the yield gain under on-farm conditions and the yield gains due to both genetic improvement and historical climate change. The calculations were done for each case study region and for the period 1994–2016 only to assess the consistency of the results across regions over the same time period. Trends in recommended N application rates for wheat are summarized in Supplementary Fig. 5. As the analysis focused on high-yielding environments only^24^, where agronomic management is close to optimal^41^, our estimates of yield gains due to agronomic management probably reflect a conservative contribution of agronomy to the wheat yield plateau.

A Yg analysis was further conducted with WOFOST for 141 field–year combinations in Flevoland, central Netherlands, to identify agronomic constraints to on-farm wheat yields. This dataset refers to a subset of field–year combinations for the entire country and spans over three growing seasons, starting in 2014–2015^21^. For each field–year combination, crop yield and detailed management data on sowing date, harvest date and fertilization dates, types and amounts were available to simulate Yp, Yw and Ywn with WOFOST^6^. The performance of the model in simulating crop growth under N-limited conditions of different experimental datasets collected in the Netherlands at different locations and years is summarized in Supplementary Table 1. The simulations conducted for the sample of farm fields used the same crop parameters used in the long-term simulations of Yp and Yw for the modern cultivar. The difference between Yp and Yw isolates the contribution of water stress to the overall Yg. The difference between Yw and Ywn isolates the contribution of suboptimal N management in farmers’ fields to the overall Yg. Finally, the difference between Ywn and Ya reflects the contribution of other management factors besides water and N management to the overall Yg. The reported N management practices were further analysed following the guidelines of the EU N Expert Panel^50^ to determine whether current N application rates were adequate to avoid N limitations on wheat yields on-farm (see Supplementary Fig. 5 and ref. ^6^ for further details).

### Reporting summary

Further information on research design is available in the Nature Portfolio Reporting Summary linked to this article.

## Supplementary information

Supplementary Information Supplementary information on model description and performance, sensitivity analysis results, impact of weather extremes and trends in nitrogen application rates.

Reporting Summary

## Source data

Source Data Fig. 1 Wheat yield trends from FAOSTAT.

Source Data Fig. 2 Wheat yield trends from crop model outputs, variety trials and province statistics.

Source Data Fig. 3 Yield response to weather variables for three regions in the Netherlands.

Source Data Fig. 4 Influence of anthesis date and grain-filling days on radiation intercepted and yield.

Source Data Fig. 5 Average yields and yields in absolute (t ha^−1^) and relative terms (percentage Yp).

## Data Availability

Data on wheat yields simulated using the WOFOST crop model (including the weather data used in the simulations), obtained in cultivar trials and reported by regional statistical authorities, are available via Zenodo at 10.5281/zenodo.17589678 (ref. ^51^). Farm field data cannot be publicly disclosed owing to privacy reasons. Source data are provided with this paper.

## Code availability

The source code of the WOFOST crop model is available via GitHub at https://github.com/ajwdewit/pcse. The source code of the development version of the model used in the simulations and the scripts developed for data analysis are available via GitHub at https://github.com/jvasco323/nfood-yield-plateau.
