# Supplementary material for: Agronomic management drives the wheat yield plateau in high-yielding environments of northwest Europe
Source: Nat Food. 2026 Jan 20;7(1):45–54. doi: 10.1038/s43016-025-01286-w (PMC12851936; doi:10.1038/s43016-025-01286-w)
Supplement: Supplementary file 1 — Supplementary information on model description and performance, sensitivity analysis results, impact of weather extremes and trends in nitrogen application rates. [file 43016_2025_1286_MOESM1_ESM.pdf]

nature food

Supplementary information https://doi.org/10.1038/s43016-025-01286-w

Agronomic management drives the wheat yield plateau in high-yielding environments of northwest Europe

In the format provided by the authors and unedited

Table of contents

1. Description and performance of the WOFOST crop model

2. Wheat yield gains due to historical climate change and improved agronomy across yield levels, soil types, and wheat varieties

3. Summary of extreme weather events and impact of drought stress on wheat yield gains due to historical climate change

4. Trends in nitrogen application rates in the Netherlands and in the study regions

References

1. Description and performance of the WOFOST crop model

1.1. Context

The WOFOST crop growth model (de Wit et al., 2019) was calibrated and evaluated against experimental data and further used to simulate long-term changes in wheat yield, growth, and development. Simulated wheat yields were compared to those recorded in official variety trials under optimal conditions and to those reported in national statistics. Finally, crop model outputs were processed to explain the long-term variability in Yp due to historical climate variability and change and to conduct a yield gap analysis for a sample of farm fields in a high-yielding environment in the Netherlands.

1.2. Model description

The crop model results presented in this study were obtained with the WOFOST crop model as implemented in the Python Crop Simulation Environment (PCSE; de Wit et al., 2019). WOFOST is a semi-deterministic crop growth simulation model of physiological processes, including crop phenology, light interception, photosynthesis (i.e., assimilation of carbohydrates), respiration, assimilate partitioning, leaf area dynamics, evapotranspiration, among others. WOFOST simulates the potential yield (Yp) and the water-limited potential yield (Yw) with a daily time step (de Wit et al., 2019) and recent efforts extended the model to further simulate water-and-nitrogen (N)-limited yield (Ywn; see Berghuijs et al., 2024, for a full description of the model extensions). In summary, the model was extended with a layered soil mineral N balance, a crop N balance, a N stress module, a new, layered soil water model, and a new reallocation model. Another important change is that the maximum gross CO_2 assimilation rate is now calculated for all production levels from the leaf N concentration, instead of from a table function of development stage.

The growth-defining factors considered to simulate Yp include temperature, daylength, solar radiation and a set of crop parameters describing leaf area dynamics, assimilation characteristics, and dry matter partitioning. Daily crop growth is estimated as the difference between the daily gross CO_2 assimilation rate and the respiration rate, which is then partitioned to the different crop organs (i.e., roots, stems, leaves and grain) using partitioning coefficients specified according to the development stage of the crop. Daily gross CO_2 assimilation rate is calculated from the absorbed solar radiation assuming a photosynthesis light response curve of individual leaves and integrating the instantaneous photosynthesis rate through canopy depth and over the course of the day. The development stage is calculated by integrating the daily development rate over time, which is a function of temperature, vernalization state, and daylength (Ceglar et al., 2019). For Yw, the soil moisture content determines whether crop growth is limited by water stress during the simulation. This is determined through a soil water balance applying an approach based on the matric flux potential (Pinheiro et al., 2017) with a layered soil profile. The soil water balance considers rainfall and irrigation as inputs and water losses by surface runoff, soil evaporation, crop transpiration and downward percolation as outputs. Re-circulation of water in the soil profile is also considered, but water flows due to capillary rise from groundwater are not simulated (Berghuijs et al., 2024). Soil evaporation and crop transpiration are estimated based on the potential evapotranspiration and considering both soil moisture content and light interception in the canopy. Reduction in growth by water limitation occurs in

parallel to the reduction in actual transpiration relative to potential transpiration. Procedures for model calibration and evaluation are summarized below.

Actual atmospheric CO_2 concentration affects (1) the maximum gross CO_2 assimilation rate, (2) the initial light use efficiency, and (3) the potential transpiration rate in WOFOST. The maximum gross CO_2 assimilation rate is corrected for CO_2 by multiplying a reference maximum CO_2 assimilation rate (i.e., under reference CO_2 level and optimum temperature) with a modification factor that is dependent on the actual CO_2 level (see code here and here). The actual initial light use efficiency and the actual potential transpiration are calculated in a similar way (see code here and here, respectively).

1.3. Model calibration and evaluation

WOFOST was calibrated and evaluated on the Groot & Verberne (1991) data set, for which detailed data on crop dry matter, crop N uptake, and soil water and N dynamics were available, at different N fertilizer rates, for winter wheat cultivar Arminda cultivated across three locations during the 1982-1983 and 1983-1984 growing seasons. For each location x year, the phenology parameters TSUM1 (thermal time between emergence and anthesis in absence of vernalization or photoperiodicity) and TSUM2 (thermal time between anthesis and maturity) were calculated using the local measurements of minimum and maximum temperature, the Zadoks scale phenology observations, parameters for sensitivity to vernalization status and daylength sensitivity for the Netherlands (Ceglar et al., 2019), and the phenology model of WOFOST. Crop parameters that describe the relationship between leaf N concentration and maximum gross CO_2 assimilation rate in C3 crops were adopted from van Keulen & Seligman (1987) and Peng et al. (1995). The tabular functions for biomass partitioning to aboveground organs were estimated by fitting the Berghuijs et al. (2020) biomass partitioning model to the observations on dry matter partitioning over the growing seasons. Reallocation parameters, initial dry weight (TDWI), and the reference maximum rate of gross CO_2 assimilation (AMAX REF) were estimated simultaneously with the biomass partitioning functions following ten Den et al. (2022) and Berghuijs et al. (2024).

WOFOST was also calibrated and evaluated against the data set from the field experiment conducted in Wageningen during the 2013-2014 and 2014-2015 growing seasons described by Berghuijs et al. (2023). The field experiment was conducted in Wageningen under optimal conditions to evaluate the yield potential of wheat cultivars under three N fertilization levels (Berghuijs et al., 2023). The experiment was irrigated twice in 2013-2014 to avoid water stress at the end of the growing season and no irrigation was necessary in 2014-2015 due to even distribution of rainfall throughout the growing season. Crop phenology was recorded in the Feekes scale and destructive measurements of aboveground dry matter, and its partitioning to the different organs, and leaf area index were taken throughout the growing season to build a database of field observations for crop modeling purposes. N uptake and partition to crop organs was not collected during the growing season for this experiment, hence it could only be used to evaluate the simulations of Ywn at harvest time. The experiment was laid out as a randomized complete block design with three wheat cultivars with different years of release (Julius in 2009, Tabasco in 2008, and Ritmo in 1992) and three high N fertilization rates (180, 240, and 300 kg N ha^{-1}), replicated four times. Data from this experiment were used to calibrate and improve the WOFOST crop model for modern wheat varieties. All crop parameters used for these simulations were the same as calibrated on the Groot & Verberne (1991) data set, except TSUM1 (temperature

sum between emergence and anthesis), TSUM2 (temperature sum between anthesis and maturity), and AMAX_REF (maximum gross CO_2 assimilation rate under reference conditions). TSUM1 and TSUM2 were re-estimated (Berghuijs et al., 2023) to capture that the modern wheat cultivars have slightly different phenology than the old cultivar Arminda released in 1977 (TSUM1 of Arminda = 886°C d and of Julius = 880°C d; TSUM2 of Arminda = 860°C d and of Julius = 890°C d). AMAX_REF was increased from Arminda (35 kg CO_2 h^{-1} ha^{-1}) to Julius (55 kg CO_2 h^{-1} ha^{-1}) to account for slightly higher light use efficiency of the modern wheat variety (see Berghuijs et al., 2023 for further details), regardless of atmospheric CO_2 level and climate change.

1.4. Model performance against experimental data

WOFOST simulated aboveground dry matter, grain dry matter, and aboveground N uptake relatively well when compared to experimental data from Verbene & Groot (1991) (Supplementary Figure S1). The same is true for other state variables and experimental datasets, as summarized in Supplementary Table S1.

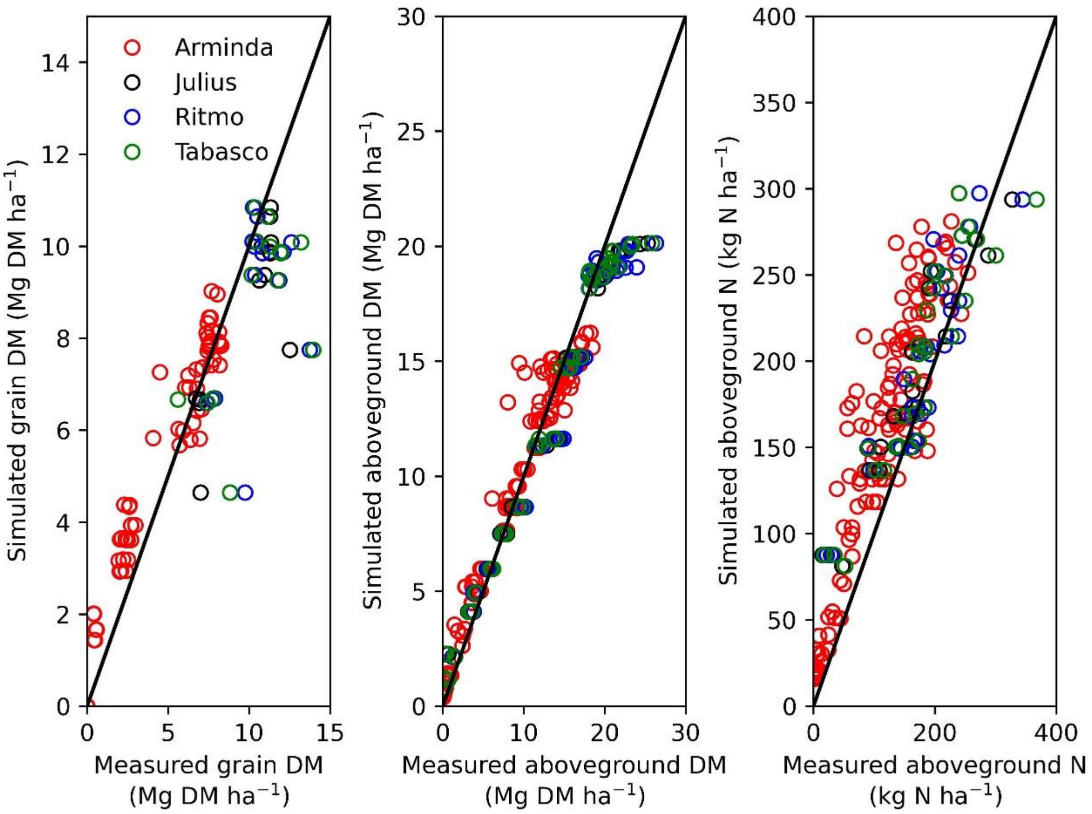

Supplementary Figure 1 | Measured and simulated total aboveground biomass, grain yield, and aboveground N uptake for wheat in the Netherlands (measurements for Arminda from Verberne & Groot, 1991; measurements for the other varieties from Berghuijs *et al.*, 2023). Crop model simulations were conducted with the crop model WOFOST.

Supplementary Table 1 | Evaluation of model performance (R^2) for selected state variables pertaining to biomass allocation and nitrogen (N) uptake at physiological maturity. Model evaluation was conducted for two experimental datasets testing wheat development and growth for different N application rates across different sites in the Netherlands, namely Groot & Verbene (1991) and Berghuijs et al. (2023). See Berghuijs et al. (2024) for further details. Abbreviations: LAI = Leaf area index, TAGB = total aboveground biomass, WLV = total weight of leaves, WST = total weight of stems, WSO = total weight of grains, NAGB = N uptake in aboveground biomass, NLV = N uptake in leaves, NST = N uptake in stems, NSO = N uptake in grains.

Year	Location	Treatment	Variety	LAI	TAGB	WLV	WST	WSO	NAGB	NLV	NST	NSO
1983	De Bouwing	N1	Arminda	0.90	0.99	0.92	0.97	0.96	0.97	0.98	0.72	0.98
1983	De Bouwing	N2	Arminda	0.88	0.99	0.90	0.95	0.98	0.98	0.91	0.93	0.97
1983	De Bouwing	N3	Arminda	0.82	0.99	0.86	0.96	0.97	0.93	0.74	0.30	0.96
1983	De Eest	N1	Arminda	0.51	0.99	0.11	0.94	0.99	0.91	0.78	0.70	0.99
1983	De Eest	N2	Arminda	0.79	0.98	0.80	0.92	1.00	0.91	0.87	0.79	0.98
1983	De Eest	N3	Arminda	0.74	0.98	0.72	0.91	0.99	0.86	0.71	0.23	0.95
1983	PAGV	N1	Arminda	0.75	0.99	0.73	0.98	0.99	0.83	0.88	0.75	0.95
1983	PAGV	N2	Arminda	0.78	1.00	0.81	0.98	0.99	0.84	0.79	0.61	0.95
1983	PAGV	N3	Arminda	0.85	1.00	0.89	0.97	0.99	0.92	0.68	0.13	0.98
1984	De Bouwing	N1	Arminda	0.94	1.00	0.96	0.99	0.99	0.97	0.83	0.91	0.98
1984	De Bouwing	N2	Arminda	0.94	0.99	0.97	0.98	0.98	0.93	0.77	0.78	0.94
1984	De Bouwing	N3	Arminda	0.97	0.99	0.97	0.98	0.97	0.93	0.81	0.75	0.95
1984	De Eest	N1	Arminda	0.90	0.99	0.96	0.97	0.98	0.97	0.94	0.89	0.99
1984	De Eest	N2	Arminda	0.94	0.99	0.96	0.97	0.96	0.91	0.61	0.87	0.98
1984	De Eest	N3	Arminda	0.94	0.99	0.97	0.97	0.98	0.95	0.76	0.87	0.98
1984	PAGV	N1	Arminda	0.76	0.90	0.89	0.78	0.95	0.90	0.91	0.90	0.94
1984	PAGV	N2	Arminda	0.80	0.98	0.96	0.86	0.96	0.96	0.93	0.94	0.97
1984	PAGV	N3	Arminda	0.77	0.99	0.93	0.83	0.96	0.96	0.92	0.89	0.96
2014	Wageningen	N1	Julius	0.96	1.00	0.96	0.97	0.90	0.93	*na*[2]	*na*[2]	*na*[2]
2014	Wageningen	N2	Julius	0.98	1.00	0.98	0.98	0.96	0.93	*na*[2]	*na*[2]	*na*[2]
2014	Wageningen	N3	Julius	0.97	1.00	0.99	0.97	0.99	0.97	*na*[2]	*na*[2]	*na*[2]
2014	Wageningen	N1	Ritmo	0.87	1.00	0.90	0.95	0.94	0.82	*na*[2]	*na*[2]	*na*[2]
2014	Wageningen	N2	Ritmo	0.94	0.99	0.95	0.95	0.92	0.87	*na*[2]	*na*[2]	*na*[2]
2014	Wageningen	N3	Ritmo	0.92	0.99	0.96	0.96	0.95	0.95	*na*[2]	*na*[2]	*na*[2]
2014	Wageningen	N1	Tabasco	0.92	1.00	0.93	0.95	0.98	0.87	*na*[2]	*na*[2]	*na*[2]
2014	Wageningen	N2	Tabasco	0.95	1.00	0.95	0.98	0.91	0.83	*na*[2]	*na*[2]	*na*[2]
2014	Wageningen	N3	Tabasco	0.96	0.99	0.98	0.95	0.81	0.95	*na*[2]	*na*[2]	*na*[2]
2015	Wageningen	N1	Julius	0.78	0.99	0.87	0.99	*na*[1]	0.89	*na*[2]	*na*[2]	*na*[2]
2015	Wageningen	N2	Julius	0.84	0.99	0.97	0.99	*na*[1]	0.93	*na*[2]	*na*[2]	*na*[2]
2015	Wageningen	N3	Julius	0.83	0.99	0.90	0.98	0.64	0.96	*na*[2]	*na*[2]	*na*[2]
2015	Wageningen	N1	Ritmo	0.77	0.99	0.94	1.00	*na*[1]	0.93	*na*[2]	*na*[2]	*na*[2]
2015	Wageningen	N2	Ritmo	0.79	1.00	0.95	1.00	*na*[1]	0.97	*na*[2]	*na*[2]	*na*[2]
2015	Wageningen	N3	Ritmo	0.79	0.99	0.89	0.97	0.56	0.93	*na*[2]	*na*[2]	*na*[2]
2015	Wageningen	N1	Tabasco	0.77	0.99	0.88	1.00	*na*[1]	0.87	*na*[2]	*na*[2]	*na*[2]
2015	Wageningen	N2	Tabasco	0.77	0.99	0.93	0.99	*na*[1]	0.91	*na*[2]	*na*[2]	*na*[2]
2015	Wageningen	N3	Tabasco	0.82	0.99	0.88	0.97	0.70	0.93	*na*[2]	*na*[2]	*na*[2]

[1] Measurements only available at harvest, hence R^2 was not computed.

[2] Data on nitrogen uptake of individual plant organs were not available.

2. Wheat yield gains due to historical climate change and improved agronomy across yield levels, soil types, and wheat varieties

Supplementary Table 2 | Underlying genetic, environment, and management factors to the yield gains attributed to genetic improvement, historical climate change, and agronomic management for wheat in Northwest Europe.

Yield trend	Data	Method	Underlying factors
**Actual farm yields**	Official statistics	Linear regression	Full Genotype x Environment x Management interactions.
**Genetic improvement (G)**	Variety trials	2-step linear regression	Variety characteristics conferring higher yield potential (e.g., crop phenology, light use efficiency).
**Historical climate change (E)**	Weather data	Crop modelling	Radiation, temperature, rainfall, ET, and atmospheric CO_2. Extreme weather events (drought, flood, frost).
**Agronomic management (M)**	Farm field data	Difference method	Field and farm level factors affecting yield losses to water and nutrient availability and pest, disease, and weed pressure.

Supplementary Table 3 | Wheat yield gains to historical climate change and agronomic management for old (Arminda released in 1977) and modern (Julius released in 2009) wheat varieties across three high-yielding environments for wheat production in Northwest Europe. Wheat yield gains to historical climate change were estimated through linear regression fitted to the potential and water-limited yields simulated for each site x variety against time. Wheat yield gains to agronomic management were estimated using the difference method considering the yield gains to historical climate change reported in this table and the yield gains due to genetic improvement and yield gains in actual yield trends depicted in Figure 2 of the main manuscript.

	Eelde (Northeast)		De Bilt (Central)		Vlissingen (Southwest)	
	**Climate (kg ha^{-1} yr^{-1})**	**Agronomy (kg ha^{-1} yr^{-1})**	**Climate (kg ha^{-1} yr^{-1})**	**Agronomy (kg ha^{-1} yr^{-1})**	**Climate (kg ha^{-1} yr^{-1})**	**Agronomy (kg ha^{-1} yr^{-1})**
**Potential yield (Yp)**						
Julius	55	-101	61	-90	50	-122
Arminda	62	-108	65	-94	57	-129
**Water-limited yield (Yw), clay soil**						
Julius	53	-99	59	-88	49	-121
Arminda	61	-107	65	-94	57	-129
**Water-limited yield (Yw), sandy soil**						
Julius	43	-89	48	-77	39	-111
Arminda	54	-100	58	-87	50	-122
**Summary statistics**						
Maximum	62	-89	65	-77	57	-111
Mean	55	-101	59	-88	50	-122
Minimum	43	-108	48	-94	39	-129

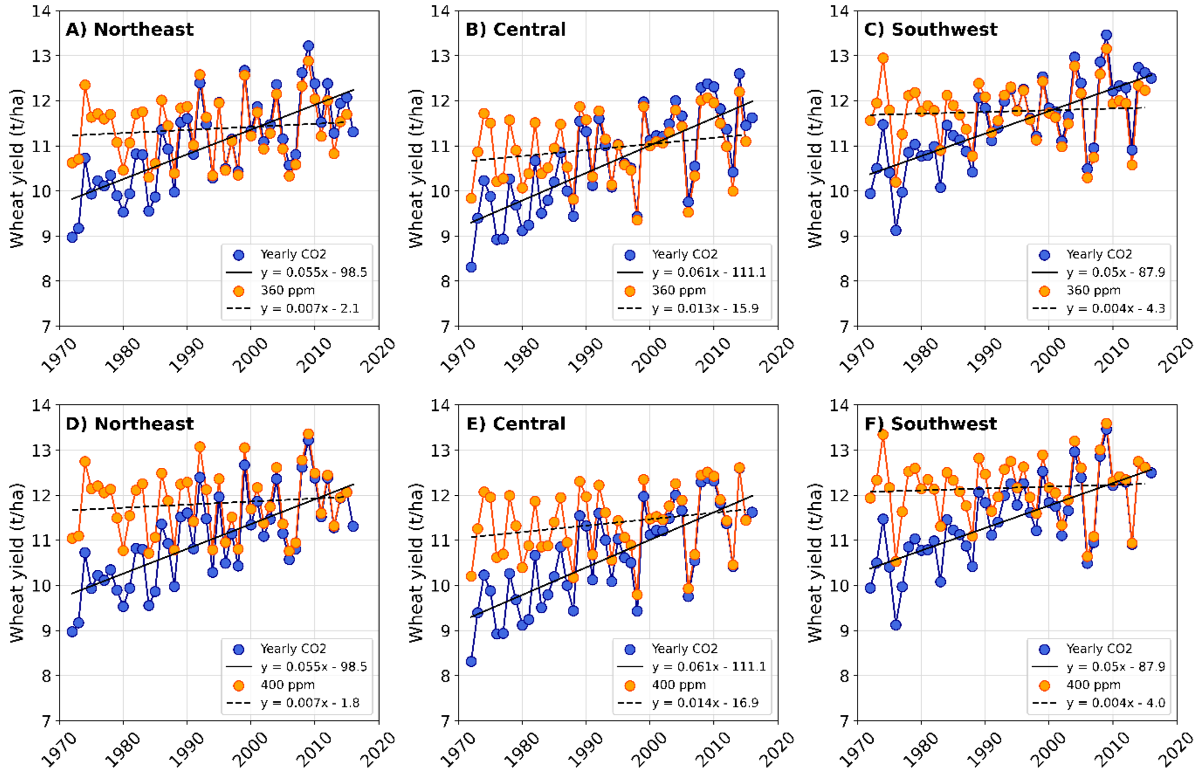

Supplementary Figure 3 | Wheat yield gains due to historical climate change across high-yielding environments in the Netherlands simulated with the WOFOST crop model for a modern wheat variety. Simulations were conducted with and without CO_2 fertilization effects on wheat yield. Two constant CO_2 concentrations were considered: (A)-(C) 360 ppm observed around the year 1994 and (D)-(F) 400 ppm observed around the year 2016. The effects of annual CO_2 fertilization are presented in all panels for comparative purposes (see also Figure 2 of the manuscript). Simulations assumed constant genetics and agronomic management throughout the simulation period (see Methods). Solid lines display linear regressions ($p < 0.001$) fitted to the data considering CO_2 fertilization effects. Dashed lines display linear regressions ($p > 0.05$) fitted to the data considering constant CO_2 of 360 and 400 ppm.

3. Summary of extreme weather events and impact of drought stress on wheat yield gains due to historical climate change

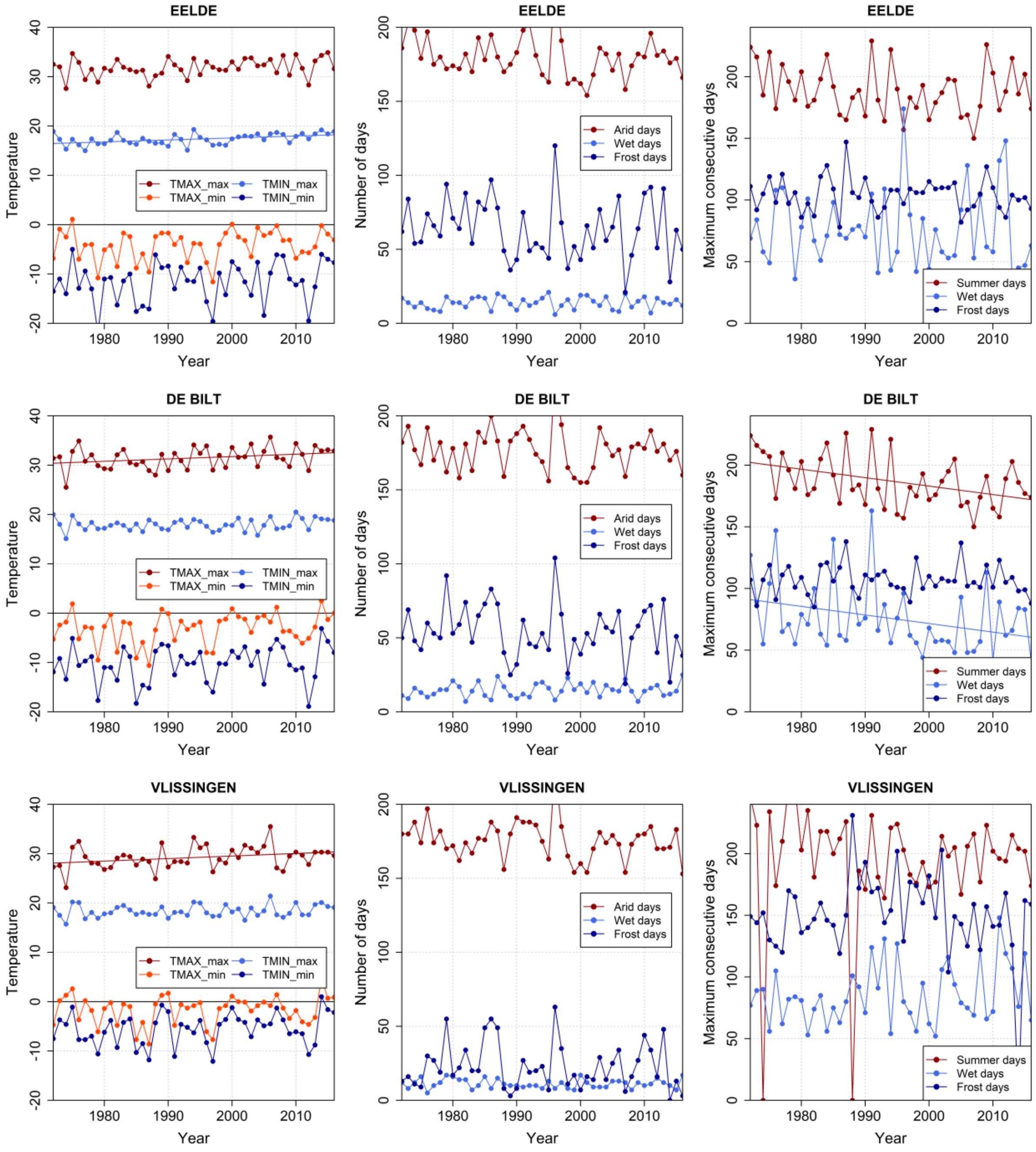

Supplementary Figure 4 | Historical trends in extreme weather events during the wheat growing season for three high-yielding environments for wheat production in Northwest Europe. Extreme events refer to: maximum of maximum and minimum temperatures, minimum of maximum and minimum temperatures, number of arid days (rainfall lower than the reference evapotranspiration), number of wet days (rainfall above 1mm), number of frost days (minimum temperature below 0°C), maximum consecutive number of summer days (maximum temperature above 30°C), maximum consecutive number of wet days (rainfall above 1mm), and maximum consecutive number of frost days (minimum temperature below 0°C). Solid lines display linear regressions for which the slope coefficient is statistically different from 0 at 5% significance level. Data refer to measured weather data from KNMI, the Royal Netherlands Meteorological Institute.

4. Trends in nitrogen application rates in the Netherlands and in the study regions

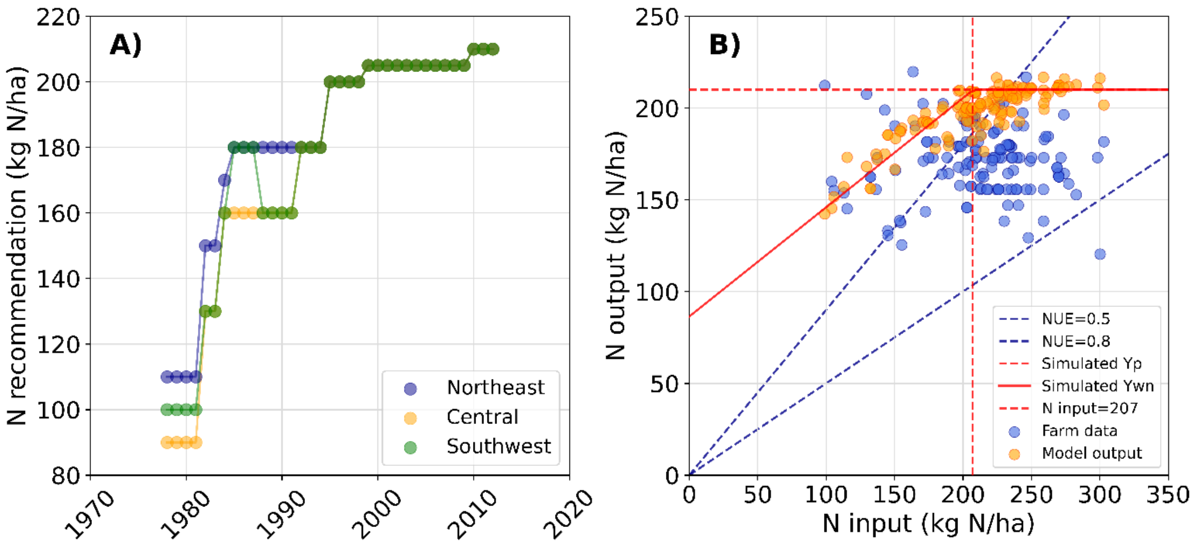

Supplementary Figure 5 | Recommended N application rates in the case study regions (A) and nitrogen use efficiency for farm-year combinations in Flevoland province, Central Netherlands (B). Recommended N application rates over time were obtained from the "KWIN" tables (see https://shop.wur.nl/kwin_en/). The farmer field data (B) was obtained from Berghuijs et al. (2024) and presents the underlying N input data for the farm yields presented in Figure 5 of the manuscript, with N input and N output estimated following the guidelines of the European N Expert Panel (EUNEP, 2015). In (B), blue dots refer to on-farm data, orange dots to the respective wheat yield (and N output) simulated with WOFOST considering the actual N management in each of the fields, solid orange line depicts the fitted response of N output to N input, the dashed orange horizontal line depicts the N output referring to the simulated potential yield, the dashed vertical orange line depicts the N input after which N becomes non-limiting for wheat productivity, and dashed blue lines indicate an N-use efficiency of 0.5 and 0.9 kg N kg^{-1} N.

References

Berghuijs et al. 2020. Identification of species traits enhancing yield in wheat-faba bean intercropping: development and sensitivity analysis of a minimalist mixture model. Plant and Soil, 455, 203-226.

Berghuijs et al. 2023. Catching-up with genetic progress: Simulation of potential production for modern wheat cultivars in the Netherlands. Field Crops Research, 296, 108891.

Berghuijs et al. 2024. Expanding the WOFOST crop model to explore options for sustainable nitrogen management: A study for winter wheat in the Netherlands. European Journal of Agronomy, 154, 127099

Ceglar et al. 2019. Improving WOFOST model to simulate winter wheat phenology in Europe: evaluation and effects on yield. Agricultural Systems, 168, 168-180.

ten Den et al. 2022. Modelling potential potato yields: Accounting for experimental differences in modern cultivars. European Journal of Agronomy, 137, 126510.

EU Nitrogen Expert Panel (EUNEP). 2015. Nitrogen Use Efficiency (NUE) - an indicator for the utilization of nitrogen in agriculture and food systems. Wageningen: Wageningen University, Alterra.

Groot & Verberne. 1991. Response of wheat to nitrogen fertilization: a data set to validate simulation models for simulation models for nitrogen dynamics in the soil. Fertiliser Research, 27, 349-383.

Keulen & Seligman. 1987. Simulation of water use, nitrogen nutrition and growth of a wheat spring crop. Wageningen, The Netherlands: Pudoc Wageningen.

Peng et al. 1995. Relationship between leaf photosynthesis and nitrogen content of field-grown rice in tropics. Crop Science, 35, 1627-1630.

Pinheiro et al. 2017. A Matric Flux Potential Approach to Assess Plant Water Availability in Two Climate Zones in Brazil. Vadose Zone Journal, 17, 1-10.

de Wit et al. 2019. 25 years of the WOFOST cropping systems model. Agricultural Systems, 168, 154-167.
