# Supplementary material for: Agronomic management drives the wheat yield plateau in high-yielding environments of northwest Europe
Source: Nat Food. 2026 Jan 20;7(1):45–54. doi: 10.1038/s43016-025-01286-w (PMC12851936; doi:10.1038/s43016-025-01286-w)
Supplement: Supplementary file 2 — Reporting Summary [file 43016_2025_1286_MOESM2_ESM.pdf]

nature portfolio

Corresponding author(s): Joao Vasco Silva

Last updated by author(s): Sep 19, 2024

Reporting Summary

Nature Portfolio wishes to improve the reproducibility of the work that we publish. This form provides structure for consistency and transparency in reporting. For further information on Nature Portfolio policies, see our Editorial Policies and the Editorial Policy Checklist.

Statistics

For all statistical analyses, confirm that the following items are present in the figure legend, table legend, main text, or Methods section.

n/a	Confirmed	
☐	☒	The exact sample size (*n*) for each experimental group/condition, given as a discrete number and unit of measurement
☒	☐	A statement on whether measurements were taken from distinct samples or whether the same sample was measured repeatedly
☐	☒	The statistical test(s) used AND whether they are one- or two-sided<br>*Only common tests should be described solely by name; describe more complex techniques in the Methods section.*
☒	☐	A description of all covariates tested
☒	☐	A description of any assumptions or corrections, such as tests of normality and adjustment for multiple comparisons
☐	☒	A full description of the statistical parameters including central tendency (e.g. means) or other basic estimates (e.g. regression coefficient) AND variation (e.g. standard deviation) or associated estimates of uncertainty (e.g. confidence intervals)
☐	☒	For null hypothesis testing, the test statistic (e.g. F, t, r) with confidence intervals, effect sizes, degrees of freedom and P value noted<br>*Give P values as exact values whenever suitable.*
☒	☐	For Bayesian analysis, information on the choice of priors and Markov chain Monte Carlo settings
☒	☐	For hierarchical and complex designs, identification of the appropriate level for tests and full reporting of outcomes
☒	☐	Estimates of effect sizes (e.g. Cohen's *d*, Pearson's *r*), indicating how they were calculated

Our web collection on statistics for biologists contains articles on many of the points above.

Software and code

Policy information about availability of computer code

Data collection	N/A
Data analysis	All analyses were conducted with open source software in R and Python. The code used for data analysis is available at https://github.com/jvasco323/nfood-yield-plateau

For manuscripts utilizing custom algorithms or software that are central to the research but not yet described in published literature, software must be made available to editors and reviewers. We strongly encourage code deposition in a community repository (e.g. GitHub). See the Nature Portfolio guidelines for submitting code & software for further information.

Data

Policy information about availability of data

All manuscripts must include a data availability statement. This statement should provide the following information, where applicable:

- Accession codes, unique identifiers, or web links for publicly available datasets
- A description of any restrictions on data availability
- For clinical datasets or third party data, please ensure that the statement adheres to our policy

Data on wheat yields simulated with the WOFOST crop model (incl. the weather data used in the simulations), obtained in variety trials and reported by regional statistical authorities are available on public repository at https://doi.org/10.5281/zenodo.17589678. Farm field data cannot be publicly disclosed due to privacy reasons.

Human research participants

Policy information about studies involving human research participants and Sex and Gender in Research.

Reporting on sex and gender	not applicable
Population characteristics	not applicable
Recruitment	not applicable
Ethics oversight	not applicable

Note that full information on the approval of the study protocol must also be provided in the manuscript.

Field-specific reporting

Please select the one below that is the best fit for your research. If you are not sure, read the appropriate sections before making your selection.

☐ Life sciences ☐ Behavioural & social sciences ☒ Ecological, evolutionary & environmental sciences

For a reference copy of the document with all sections, see nature.com/documents/nr-reporting-summary-flat.pdf

Ecological, evolutionary & environmental sciences study design

All studies must disclose on these points even when the disclosure is negative.

Study description	We used a well validated crop model to simulate wheat yields over the past half century and quantify the contribution of past climate change to wheat yield progress. We analyzed historical variety trial data to quantify the contribution of genetic yield progress to past wheat yield progress. We difference method we quantify the contribution of agronomy to past wheat yield progress. Finally, we conducted a yield gap analysis to identify the agronomic constraints to wheat productivity in recent years.
Research sample	Weather data was obtained from the Dutch Meteorological Agency. Official statistical data was obtained from the FAO and from the official statistics from the Netherlands. Variety trial data was obtained over many years from breeders in the country. Farmer field data was obtained through a partnership with a commercial company and consolidated as part of a research project.
Sampling strategy	We conducted crop model simulations for different sites to account regional (spatial) differences in results. For the yield gap analysis, we focused in a region where agronomy is close to optimal so that our results thus offer a lower bound for the importance of agronomic constraints on wheat yield.
Data collection	Results rely on crop model simulations, variety trial data, farmer field data, and official statistical data. For crop model simulations, weather data was obtained from the Dutch Meteorological Agency. Official statistical data was obtained from the FAO and from the official statistics from the Netherlands. Variety trial data was obtained over many years from breeders in the country. Farmer field data was obtained through a partnership with a commercial company and consolidated as part of a research project.
Timing and spatial scale	Results refer to the Nortwest Europe over the period 1972-2016.
Data exclusions	No data were excluded from the analysis.
Reproducibility	All data manipulation and analysis were conducted with reproducible R and Python scripts.
Randomization	This is not relevant to this study since we rely on crop model simulations and relatively large scales.
Blinding	Blinding was not relevant for this study given its crop modeling focus.

Did the study involve field work? ☐ Yes ☒ No

Reporting for specific materials, systems and methods

We require information from authors about some types of materials, experimental systems and methods used in many studies. Here, indicate whether each material, system or method listed is relevant to your study. If you are not sure if a list item applies to your research, read the appropriate section before selecting a response.

Materials & experimental systems

n/a	Involved in the study
☒	☐ Antibodies
☒	☐ Eukaryotic cell lines
☒	☐ Palaeontology and archaeology
☒	☐ Animals and other organisms
☒	☐ Clinical data
☒	☐ Dual use research of concern

Methods

n/a	Involved in the study
☒	☐ ChIP-seq
☒	☐ Flow cytometry
☒	☐ MRI-based neuroimaging
